# Transglutaminase 2 affinity and enzyme‐substrate intermediate stability as determining factors for T‐cell responses to gluten peptides in celiac disease

**DOI:** 10.1002/eji.202249862

**Published:** 2022-07-13

**Authors:** Sunniva F. Amundsen, Jorunn Stamnaes, Marie Fleur du Pré, Ludvig M. Sollid

**Affiliations:** ^1^ KG Jebsen Coeliac Disease Research Centre Institute of Clinical Medicine University of Oslo Oslo Norway; ^2^ Department Immunology Oslo University Hospital Oslo Norway

**Keywords:** autoimmunity, B cells, celiac disease, T cells, transglutaminase 2

## Abstract

The adaptive immune response of celiac disease (CeD) involves presentation of gluten peptides to CD4^+^ T cells by transglutaminase 2 (TG2) specific B cells. This B‐cell/T‐cell crosstalk is facilitated by involvement of TG2:gluten peptide complexes that act principally in the form of enzyme‐substrate intermediates. Here, we have addressed how gluten peptide affinity and complex stability in the presence of secondary substrates affect the uptake of TG2:gluten peptide complexes by TG2‐specific B cells and the activation of gluten‐specific T cells. We studied affinity of various gluten peptides for TG2 by biochemical assay, and monitored uptake of gluten peptides by TG2‐specific B cells by flow cytometry. Crosstalk between TG2‐specific B cells and gluten‐specific T cells was assayed with transfectants expressing antigen receptors derived from CeD patients. We found that gluten peptides with high TG2 affinity showed better uptake by TG2‐specific B cells. Uptake by B cells, and subsequent activation of T cells, was negatively affected by polyamines acting as secondary TG2 substrates. These results show that affinity between gluten peptide and TG2 governs the selection of T‐cell epitopes via enhanced uptake of TG2:gluten complexes by TG2‐specific B cells, and that exogenous polyamines can influence the CeD immune responses by disrupting TG2:gluten complexes.

## Graphical abstract

Key immunological processes in celiac disease involve interaction between transglutaminase 2 (TG2)‐specific B cells and gluten‐specific T cells. The outcome of these processes is governed by the affinity between different gluten peptides and the enzyme (I) as well as by the presence of secondary TG2 substrates (II).

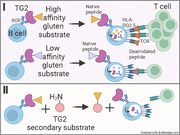

## Introduction

CeD is an autoimmune‐like disease caused by a harmful immune response to dietary gluten proteins of wheat (α‐, γ‐, ω‐gliadins, and glutenins), barley (hordeins), and rye (secalins). Patients with CeD have CD4^+^ T cells that recognize deamidated gluten peptides bound to the CeD‐associated human leukocyte antigens (HLA) molecules HLA‐DQ2.5, HLA‐DQ2.2, or HLA‐DQ8 [1]. TG2 is a central player in the pathogenesis of CeD, both as the target of disease‐specific autoantibodies and as the enzyme that catalyzes deamidation of gluten peptides, a modification that is necessary for activation of the disease‐driving gluten‐specific CD4^+^ T cells [2]. The calcium dependent TG2 enzyme targets glutamine residues in peptides and proteins through a modified ping‐pong mechanism [3]. The active site cysteine performs a nucleophilic attack on the glutamine residue side chain and forms a transient but covalent thioester enzyme‐substrate intermediate complex upon release of ammonia. In the second step, the thioester intermediate complex is subjected to nucleophilic attack from a secondary substrate where the enzyme is released, and a product is formed. If the nucleophile is a primary amine from a small molecule, peptide, or protein, a covalent amidebond is formed resulting in a transamidated product. In the absence of a primary amine, water will act as a nucleophile and the glutamine residue is converted to glutamic acid (deamidation). Because water is a poor nucleophile compared to a primary amine, collapse of the thioester intermediate complex is slower than in the presence of primary amines [4]. TG2 targets glutamine residues in proteins and peptides in a highly sequence‐specific manner with a strong preference for glutamines within the motif QXP [5, 6]. Gluten is very rich in glutamine and proline residues. Many gluten peptides are therefore excellent substrates for TG2 [5, 6]. The preferred substrates in complex gluten peptide mixtures are also the most immunodominant T‐cell epitopes of CeD [7].

The disease‐associated antibodies to TG2 are formed in subjects who carry the CeD‐associated HLA‐DQ molecules and who consume gluten [2]. This HLA and gluten dependence for antibody production can mechanistically be explained by a hapten‐carrier‐like mechanism that relies on the uptake of TG2:gluten complexes [8]. B cells need T‐cell help for antibody production, and TG2‐specific B cells will by internalization of TG2:gluten complexes receive help from gluten‐specific T cells. The TG2‐specific B cells bind the TG2:gluten complex by the cell surface B‐cell receptor (BCR), and upon antigen processing, deamidated gluten peptides are loaded onto HLA‐DQ molecules and presented to CD4^+^ T cells with gluten‐specific T‐cell receptors (TCRs).

Theoretically, two types of TG2:gluten complexes can be involved in the B‐cell/T‐cell crosstalk in CeD. A gluten peptide can be bound to the active site cysteine in the form of a thioester enzyme–substrate complex, or it can be bound via an isopeptide bond to a lysine residue on the surface of TG2 [9].  Recently, we provided evidence that pathogenic TG2 in CeD derives from shed enterocytes, and we gave evidence for the involvement of the former, but not the latter type of complex [10]. Conceivably, the amount of thioester intermediate complexes that can be taken up by the BCR of a B cell will depend on the affinity of the gluten substrate to the active site of TG2 as well as the stability of the complex. Nucleophilic attack by secondary TG2 substrates present in the environment where B cells and T cells naturally interact in the gut may thus be an important determinant. In this study, we have examined how gluten peptide affinity to TG2 affects the crosstalk between TG2‐specific B cells and gluten‐specific T cells, and we have further addressed how natural small molecule primary amines affect the B‐cell mediated presentation of gluten peptides to T cells.

## Results

### B‐cell presentation of various gluten epitopes to T cells via TG2:gluten complexes

**Figure 1:**
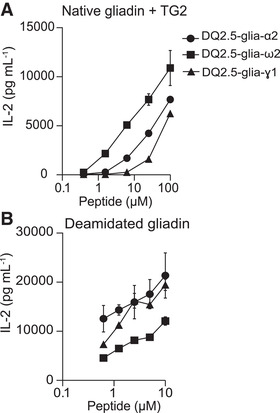
**T‐cell activation by B‐cell uptake of TG2:gluten complexes or deamidated gluten peptides**. A20 cells expressing a TG2‐specific BCR and HLA‐DQ2.5 were incubated with increasing concentrations of synthetic native or deamidated gluten peptides in the presence or absence of recombinant TG2 and CaCl_2_ followed by co‐culture with TCR transfectants specific for the respective gluten T‐cell epitopes. T‐cell activation was assessed by ELISA quantification of IL‐2 secreted into the cell culture medium. (A) Activation of TCR‐transfectant cells specific for the DQ2.5‐glia‐α2, DQ2.5‐glia‐ω2, or DQ2.5‐glia‐γ1 epitopes after co‐culture with TG2‐specific A20 cells incubated with recombinant TG2 and native gluten peptides (N‐α‐gliadin 20‐mer, N‐ω‐gliadin 19‐mer and N‐γ‐gliadin 21‐mer) in presence of CaCl_2_. (B) Activation of TCR‐transfectant cells specific for the DQ2.5‐glia‐α2, DQ2.5‐glia‐ω2, or DQ2.5‐glia‐γ1 epitopes after co‐culture with TG2‐specific A20 cells incubated with deamidated gluten peptides (D‐α‐gliadin 20‐mer, D‐ω‐gliadin 17‐mer and D‐γ‐gliadin 21‐mer). The figure shows mean of sample triplicates ± SD from one of two independent experiments.

Given the central role of TG2:gluten complexes for the presentation of gluten epitopes to T cells, we aimed to investigate whether gluten peptides that harbor different gluten T‐cell epitopes differ in their ability to generate TG2:gluten complexes. To this end, we performed B‐cell/T‐cell collaboration assays using A20 cells transfected with a TG2‐specific BCR as well as HLA‐DQ2.5. This cell line was incubated with recombinant, active TG2 and synthetic peptides representing the DQ2.5‐glia‐α2, DQ2.5‐glia‐ω2, or DQ2.5‐glia‐γ1 epitopes before the addition of TCR transfectants specific for the epitopes of the distinct peptides. The native peptides clearly differed in their ability to stimulate the specific T cells with a stimulation rank order of DQ2.5‐glia‐ω2 > DQ2.5‐glia‐α2 > DQ2.5‐glia‐γ1 (Figure 1A; Figure S1). Differential response to gluten T‐cell epitopes could conceivably be explained by different HLA binding capacities of the peptides, different TCR affinities for peptide‐HLA complexes, or different TG2 turnover rates for the generation of deamidated peptides. B‐cell/T‐cell collaboration assays using already deamidated peptides efficiently activated T cells through direct binding to DQ2.5, but did not reproduce the same rank order as incubation with native peptide and TG2 (Fig. 1B, Figure S1). These results suggest that different gluten peptide affinities for TG2 may dictate the formation of TG2:gluten complexes and thereby BCR mediated uptake by the TG2‐specific B cell and subsequent stimulation of the gluten‐specific T cells.

### Gluten peptides giving rise to T‐cell epitopes differ in TG2 affinity

**Figure 2:**
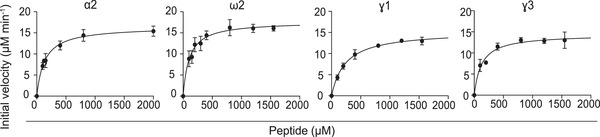
**Kinetic data for TG2 turnover of peptides harboring gluten T‐cell epitopes**. TG2 substrate properties of native 9‐mer gliadin peptides harboring the DQ2.5‐glia‐α2, DQ2.5‐glia‐ω2, DQ2.5‐glia‐γ1, and DQ2.5‐glia‐γ3 T‐cell epitopes were compared using a GDH‐coupled ammonium release assay that measures TG2:substrate thioester intermediate complex formation. The figure shows the fitted Michaelis‐Menten curves for each peptide (mean ± SD from three independent experiments per peptide).

To test the influence of substrate affinity for TG2 on B‐cell uptake, we determined kinetic parameters for gluten peptides harboring the essential TG2 targeted glutamine residue within the DQ2.5‐glia‐α2, DQ2.5‐glia‐ω2, DQ2.5‐glia‐γ1, or DQ2.5‐glia‐γ3 T‐cell epitopes. To directly compare differences in TG2 affinity between the glutamines from the different peptides, we tested 9‐mer peptides harboring only one TG2 targeted glutamine residue. We used a coupled glutamate dehydrogenase (GDH) enzymatic assay that measures the release of ammonia and hence reports on the formation of the thioester intermediate complex. Initial reaction rates and calculated kinetic parameters are shown in Figure 2 and Table 1. All four peptides showed comparable *k*_cat_ values, but differed in their affinity to TG2 (*K*_m_; Table 1). Native 9‐mer peptides representing the DQ2.5‐glia‐α2, the DQ2.5‐glia‐ω2 and DQ2.5‐glia‐γ3 epitopes had comparable *K*_m_ values, whereas the native 9‐mer peptide representing the DQ2.5‐glia‐γ1 epitope showed a twofold lower affinity to TG2 (Table 1).

**Table 1:** Summary of kinetic data from ammonia release assay. The table reports mean and SD from three independent experiments

Peptide	k_cat_ (min^–1^)	K_m_ (mM)	k_cat_/ K_m_ (min^–1^/mM)
α2	25.7±0.8	0.13±0.02	197
ω2	27.6±1.0	0.10±0.02	276
γ1	24.0±0.7	0.24±0.02	100
γ3	23.2±1.0	0.13±0.03	197

### Peptides with high affinity for TG2 are more effectively bound by TG2‐specific B cells

**Figure 3:**
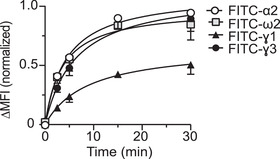
**Binding and uptake of gluten peptides by TG2‐specific B cells**. A20 cells expressing a TG2‐specific BCR (679‐14‐E06) or non‐TG2 specific BCR (693‐2‐F02) were incubated with recombinant TG2 and native FITC‐labeled α2, ω2, γ1, and γ3 9‐mer gliadin peptides in the presence of CaCl_2_. Cell‐bound FITC peptides were measured by flow cytometry. Delta (∆) MFI was calculated by subtracting MFI of non‐TG2‐specific B cells from MFI of TG2‐specific B cells. The figure shows the mean normalized delta MFI (±SD) from three independent experiments.

Next, we asked whether the level of uptake of the four different gluten peptides by a TG2‐specific B cell correlated with peptide affinity to TG2. We determined by flow cytometry the time‐dependent uptake of FITC‐conjugated synthetic gliadin 9‐mer peptides by A20 cells transfected with a TG2‐specific BCR (Figure 3; Figure S2). A20 cells transfected with a TG2 specific or non‐TG2 specific BCR were incubated with native peptides in presence of active TG2. At the indicated time points, the enzymatic activity of TG2 was stopped by the addition of EDTA, the cells were washed and fixed to remove free peptide before analysis. We found that native 9‐mer peptides harboring glutamine residues targeted by TG2 to generate the DQ2.5‐glia‐α2, DQ2.5‐glia‐ω2, and DQ2.5‐glia‐γ3 epitopes were all taken up more efficiently by the TG2 specific B cells than the peptide harboring the glutamine residue targeted by TG2 to generate the DQ2.5‐glia‐γ1 epitope. The differences in uptake largely mirrors the differences in peptide affinity for TG2 (Table 1). To address the contribution of iso‐peptide‐linked TG2:gluten complexes on peptide uptake by TG2‐specific B cells in our system, we pre‐incubated TG2 and native 9‐mer peptides for 30 min to allow for the maximal accumulation of such complexes (Figure S3). For all four peptides, we observed two to fourfold higher uptake of peptide when TG2‐specific B cells were offered enzymatically active TG2 and native gluten peptides compared to a pre‐incubated TG2:gluten mixture. These results argue that gluten peptide uptake by TG2‐specific B cells in our system predominantly occurs through the uptake of thioester enzyme–substrate complexes.

### The presence of primary amines affects gluten–peptide uptake by TG2‐specific B cells

**Figure 4:**
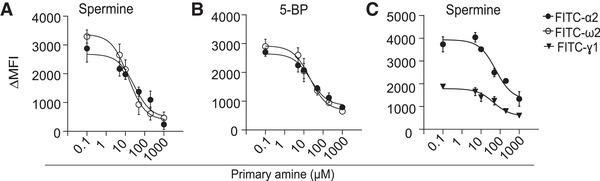
**Effect of TG2 secondary substrate on the uptake of TG2:gluten complexes by TG2‐specific B cells**. A20 cells expressing a TG2‐specific BCR (679‐14‐E06) or non‐TG2‐specific BCR (693‐2‐F02) were incubated for 10 min with recombinant TG2 and native FITC‐labeled α2, ω2, and γ1 9‐mer gliadin peptides in the presence of CaCl_2_ and increasing concentrations of primary amines. Cell‐bound FITC peptides were measured by flow cytometry and delta (∆) MFI was calculated as for Figure 3. (A) Uptake of FITC‐α2 and FITC‐ω2 in the presence of spermine. (B) Uptake of FITC‐α2 and FITC‐ω2 in the presence of 5‐BP. (C) Uptake of FITC‐α2 and FITC‐ γ1 in the presence of spermine. The figure shows mean of sample triplicates (+/‐ SD) from one of three (A,B) or one of two (C) independent experiments.

Conceivably, any factor affecting the stability of TG2:gluten complexes would also affect the uptake by TG2‐specific B cells. Nucleophilic attack of the thioester intermediate to break the thioester bond is the most relevant. We therefore addressed the impact of spermine, a physiological relevant primary amine, as well as biotinylated cadaverine (5‐BP, commonly used as a TG2 secondary substrate) on B‐cell uptake of gluten peptides. We addressed uptake of FITC‐conjugated 9‐mer native gluten peptides harboring the DQ2.5‐glia‐α2 and DQ2.5‐glia‐ω2 epitopes in the presence of increasing concentrations of primary amine (Figure 4A,B; Figure S4A,B). Indeed, TG2‐mediated uptake of FITC‐9‐mer peptides by the TG2‐specific B cell was readily inhibited by the addition of amines in a dose‐dependent manner, with average IC50 values in the range of 26–72 µM both for spermine and 5‐BP (Table S1). The presence of spermine effectively inhibited uptake of both FITC‐9’mer DQ2.5‐glia‐ω2 and DQ2.5‐glia‐γ1 epitope harboring peptides (Figure 4C; Figure S4). Thus, destabilization of the TG2 thioester intermediate by primary amines directly affects the uptake of gluten peptides by TG2‐specific B cells.

### Primary amines prevent activation of gluten‐specific T cells by TG2‐specific B cells

**Figure 5:**
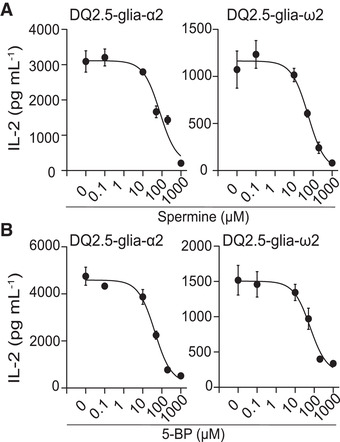
**Effect of TG2 secondary substrate on T‐cell activation via TG2‐specific B cells**. A20 cells expressing a TG2‐specific BCR and HLA‐DQ2.5 were incubated with recombinant TG2, N‐α‐gliadin 33‐mer or N‐ω‐gliadin 34‐mer peptides in presence of CaCl_2_ and increasing concentrations of spermine (A) or 5‐BP (B) followed by co‐culture with TCR transfectants specific for either the DQ2.5‐glia‐α2 or DQ2.5‐glia‐ω2 epitopes. T‐cell activation was assessed by ELISA quantification of IL‐2 secreted into the cell culture medium. The figure shows mean of sample triplicates (+/‐ SD) from one of three independent experiments.

We next addressed if the presence of primary amines through an effect on uptake of TG2:gluten complexes also affects the ability of TG2‐specific B cells to activate gluten‐specific T cells. To this end, we performed a B‐cell/T‐cell collaboration assay in the presence of spermine (Figure 5A; Figure S5) or 5‐BP (Figure 5B; Figure S5) using the highly immunogenic native α‐gliadin 33‐mer and ω‐gliadin 34‐mer peptides that harbor the immunodominant DQ2.5‐glia‐α2 and DQ2.5‐glia‐ω2 T‐cell epitopes, respectively. T‐cell stimulation was monitored as IL‐2 production by two TCR‐transfectant cells specific for these two epitopes. The presence of primary amines inhibited T‐cell stimulation in a dose‐dependent manner with IC50 inhibition values in the range of 27–58 µM, both for spermine and 5‐BP (Table S2). We observed no effect of primary amines on the T‐cell response to deamidated gluten peptides (Figure S6) that rules out off‐target effects of primary amines in our assay.

## Discussion

A key role of gluten‐specific CD4^+^ T cells in the pathogenesis of CeD is well established [1]. Being an integral part of the adaptive immune system, B cells are intimately involved in the function of T cells [11]. The presence of antibodies to TG2 are excellent predictors of CeD thus somehow suggesting the involvement of the antibodies in the pathogenesis. Mounting evidence suggests that the antibodies are involved in disease development as BCRs, the antigen receptors of B cells, and less likely as soluble effector molecules [10, 12]. Evidence also suggests that there is a link between gluten‐specific T cells and antibody production to TG2 and that this link involves TG2:gluten complexes, primarily TG2:gluten thioester intermediate complexes [10]. We here demonstrate that the affinity of gluten peptides to TG2 dictates how much TG2:gluten complexes are formed and thereby taken up by TG2‐specific B cells. This antigen uptake directly affects how much stimulatory gluten peptide is presented to gluten‐specific CD4^+^ T cells. Such a mechanism can very well explain the observation that T‐cell epitopes of gluten rank on the top among the gluten peptide that are preferred substrates for TG2 [7].

The peptide primary sequence that surrounds a glutamine residue determines whether the glutamine is targeted by TG2 [5, 6, 13], and the sequences of gluten peptides often provide unusually good fits for the active side of TG2 [14, 15]. Peptide length also affects gluten peptide turnover [14] that can explain why we see a somewhat higher T‐cell response to the DQ2.5‐glia‐ω2 epitope peptide compared to the DQ2.5‐glia‐α2 epitope peptide in our B‐cell/T‐cell assay despite their similar TG2 affinity as short 9‐mer peptides. The overall good agreement between T‐cell response and TG2 affinity for the respective core 9‐mer T cell epitopes however argues that TG2 affinity is the strongest determinant of T‐cell epitope uptake and presentation in our experimental set‐ups.

In the model with the involvement of thioester‐linked enzyme–substrate complexes, the kinetic stability of TG2:gluten peptide complex will influence the peptide antigen uptake by TG2‐specific B cells. As demonstrated here, TG2‐specific B cells will upon addition of exogenous strong nucleophiles take up less gluten peptides. The presence of secondary TG2 substrates will therefore also affect the T‐cell response when TG2‐specific B cells serve as antigen‐presenting cells. B cell uptake of TG2 in complex with gluten will *in vivo* always occur against a backdrop of protein‐bound lysine and our assays in the presence of free lysine in the cell medium. Still, we observe a clear effect of exogenously added primary amine. Thus, polyamines naturally present in the gut lumen may influence TG2:gluten complex formation and stability [16]. Gut lumen polyamines can derive from bacteria, by endogenous synthesis, or from ingested food [17, 18]. A study in rats suggested recirculation of polyamines in the gut lumen via enterohepatic circulation [19]. Spermidine is the predominant polyamine of foods, and food categories with the highest contents are cereals, legumes, and soy derivates [20]. Natural, unbranched polyamines with a length of ≥5 carbon atoms are all equally good secondary substrates for TG2 [21]. Thus, the stability of the thioester enzyme‐substrate complexes will be affected by the collective concentrations of polyamines present. The knowledge about polyamine concentrations in the gut is limited. In a study of five fasting volunteers, the total concentration of various polyamines in duodenal aspirates was estimated to around 45 µM with concentrations of spermidine and spermine of 3.2 and 4.0 µM, respectively [18]. Another study of five subjects estimated the concentration in duodenal fluid of spermidine and spermine to be 13.1 and 6.3 µM, respectively [22]. Thus, polyamine concentrations in the gut will fluctuate in the µM range depending upon the intake of foodstuff.

CeD develops as the result of an interplay between genetic and environmental factors. Gluten is a well‐known environmental factor that is obligatory for disease development. Other suspected environmental factors are microbes, particularly enteroviruses [23]. Our study raises the possibility that polyamines can be an additional environmental factor, either from ingested food or produced by gut resident bacteria. We speculate that the effect of this factor would be most critical in the initiation of the adaptive immune response prior to clonal amplification of T cells and B cells after which a high sensitivity to gluten is established. While much more data will need to be collected to draw conclusions, this raises the theoretical possibility that the development of CeD can be prevented by an exogenous supply of polyamines and also that polyamines potentially can be harnessed for therapy.

In this study, we present two main findings: we show how the affinity of gluten peptides to TG2 governs the selection of immunodominant T‐cell epitopes, and we show how polyamines by the destruction of the enzyme–substrate intermediate complexes curtail the activation of the gluten‐specific CD4^+^ T cells. These fundamental observations may have implications for our understanding of CeD including how the development of the disease can be prevented.

## Materials and methods

### Peptides

The sequences and names of four 9‐mer synthetic gluten peptides used in the study, representing the native DQ2.5‐glia‐α2, DQ2.5‐glia‐ω2, DQ2.5‐glia‐γ1, and DQ2.5‐glia‐γ3 epitopes, are given in Table 2. The sequences and names of the other synthetic gluten peptides used in the study are listed in Supplementary Table S3. Some of the 9‐mer peptides were synthesized with an N‐terminal FITC label that was linked via 6‐aminocaproic acid (Ahx). Both native and deamidated peptides were used, this is designated with “N‐” for native or “D‐” for deamidated in front of the peptide names.

**Table 2:** Synthetic 9‐mer gluten peptides harboring the TG2‐targeted glutamine residues of the DQ2.5‐glia‐α2, DQ2.5‐glia‐ω2, DQ2.5‐glia‐γ1 and DQ2.5‐glia‐γ3 epitopes. The “Ac” denotes acetylation. The glutamine/glutamate residue important for the HLA‐TCR interaction are given in bold. Residues corresponding to 9‐mer core regions of T‐cell epitopes are underlined

Name	Sequence	Source
α2	PQP **Q** LPYPQ	Genscript
ω2	PQP **Q** QPFPW	Genscript
γ1	Ac‐QQSFP **Q** QQR	Genscript
γ3	Ac‐QP **Q** QPYPQQ	Genscript

### Recombinant TG2

Recombinant human TG2 with a C‐terminal His‐tag was expressed in Sf+ express insect cells. Briefly, human *TGM2* was cloned into the pACAB3 vector, and protein was expressed for 3 days followed by lysis and purification by nickel‐nitrilotriacetic acid affinity chromatography as previously described [24].

### TG2 enzyme kinetic assay

Kinetic parameters for the 9‐mer peptides α2, ω2, γ1, and γ3 were measured using a coupled GDH catalyzed reaction assay [25] performed in microtiter plates as previously described [26]. Here, the following components were used: (1) a 5× stock MOPS buffer consisting of 1 M 3‐(N‐morpholino)propanesulfonic acid pH 7.2, 25 mM CaCl_2_, 5 mM ETDA, and 50 mM α‐ketoglutarate (Sigma Aldrich) in distilled water; (2) nicotinamide adenine dinucleotide hydrogen (NADH, Sigma–Aldrich); (3) GDH from beef liver (Roche); (4) gluten peptides; (5) recombinant TG2 solved in 1mM EDTA/Tris‐buffered saline. Components 1–4 were mixed in PCR‐strips (Axygen) to give final concentrations of 1x stock buffer, 1 mM NADH, 0.125 U/µL GDH, and various peptide concentrations. The mixture was preincubated in the dark for 15 min, and the reaction was started by the addition of recombinant TG2 (final concentration of 50 µg/mL) followed by transfer to a microtiter plate (Thermo Scientific). Absorbance (340 nm) was measured continuously for 50 min at 37°C using a Victor 3V Multi‐Label Microplate Reader (Perkin Elmer‐Wallac). Initial reaction velocities were calculated from the linear area of the absorbance curves by determining the change in absorbance over time compared to the change from an NADH standard curve. The Michaelis constants (*K*_M_) and turnover rates (*k*_cat_) were calculated using the *k*_cat_ equation analysis tool in GraphPad Prism 8.3.0 (GraphPad Software).

### BCR‐ and TCR‐transfectant cell lines

Murine A20 B lymphoma cells engineered to express either the celiac patient‐derived TG2‐specific BCR 679‐14‐E06 or the non‐TG2‐specific BCR 693‐2‐F02 as human IgD are previously described [27, 28]. For B‐cell/T‐cell collaboration assays, we used A20 BCR transfectants also transduced to express HLA‐DQ2.5 [27]. Murine BW58α^–^β^–^ cells were retrovirally transduced to express human CD4 as well as the celiac patient‐derived gluten‐specific TCR from a DQ2.5‐glia‐γ1‐reactive T‐cell clone (TCC 4.32) [29] as previously described [30]. The generation of TCR transfectants expressing DQ2.5‐glia‐α2‐reactive TCR (TCC 364.14) [30] and DQ2.5‐glia‐ω2‐reactive TCR (clone 737.30) [31] have been described previously [30, 32].

### B‐cell uptake of gluten peptides

B‐cell uptake of gluten peptides was measured essentially as previously described [10]. The following 9‐mer peptides were compared: FITC‐α2, FITC‐ω2, FITC‐γ1, and FITC‐γ3. In brief, A20 cells (300 000/well) expressing TG2‐specific (679‐14‐E06) or non‐TG2 specific BCR (693‐2‐F02) were incubated with recombinant TG2 (final 3 µg/mL), 5 µM FITC peptide, and 2 mM CaCl_2_ in RPMI at a final volume of 50 µL. Following incubation at 37°C, the TG2 enzymatic activity was quenched by the addition of 100 µL 2 mM EDTA/RPMI at specific timepoints. Cells were washed, fixed in 2% methanol‐free formaldehyde (Invitrogen), resuspended in 2% fetal calf serum (FCS)/PBS, and analyzed by flow cytometry (Attune NxT Flow Cytometer, Thermo Scientific). The gating strategy for flow cytometry analysis is shown in Figure S7. For each peptide, a delta MFI was calculated by subtracting MFI from non‐TG2 specific A20 cell (unspecific binding) from MFI of TG2‐specific A20 cells. Delta MFI was normalized for each experiment by dividing by the highest delta value per experiment. To assess the contribution and uptake of isopeptide‐linked TG2:gluten complexes, TG2 (3 µg/mL) and FITC‐labelled peptides (5 µM) were preincubated in RPMI with 2 mM CaCl2 at 37°C for 30 min before addition of A20 cells followed by 30 min incubation at 37°C. To examine the effect of TG2 secondary substrates on peptide uptake, the primary amines spermine (Sigma–Aldrich) and biotinylated cadaverine (5‐biotinamido‐pentylamine; 5‐BP, Pierce) were added to the reaction mixture (0.1–1000 µM final concentration), followed by 10 min incubation at 37°C and flow cytometry analysis. The dose‐response curve fitting to find IC50 was done using the “[Inhibitor] vs. response” equation in GraphPad Prism 8.3.0. Of note, the presence of Lysine HCl in RPMI medium represents an experimental constant in our assays that is not accounted for when calculating the competitive effect of amine.

### B‐cell/T‐cell collaboration assay

The B‐cell/T‐cell collaboration assays were set up as previously described [33]. To compare the uptake and presentation of different gluten peptides to T cells, A20 cells (5 mill/mL) expressing the TG2‐specific BCR (679‐14‐E06) and HLA DQ2.5 were incubated with recombinant TG2 (final 3 µg/mL) and varying amounts of N‐α‐gliadin 20‐mer, N‐ω‐gliadin 19‐mer, or N‐γ‐gliadin 21‐mer in RPMI supplemented with 2 mM CaCl_2_. After a 30 min incubation at 37°C the cells were washed with 1% FCS/RPMI and resuspended in 5% FCS/RPMI to give a final 1.5 million cells/mL (150 000/well) followed by 2 h incubation at 37°C. Next, TCR‐transfectant cells specific for either DQ2.5‐glia‐α2, DQ2.5‐glia‐ω2, or DQ2.5‐glia‐γ1 were added (25 000/well), followed by overnight incubation at 37°C. The next day, IL‐2 concentration was measured in the culture supernatant by ELISA as previously described [30]. The responsiveness of different TCR transfectants was assessed in the same way, but without the 30 min incubation with TG2, and using the synthetic deamidated peptides (d‐α‐gliadin 20‐mer, d‐ω‐gliadin 17‐mer, and d‐γ‐gliadin 21‐mer). To assess how TG2 secondary substrate affects peptide uptake and presentation to T cells, TG2‐specific A20 cells expressing DQ2.5 were incubated with TG2, CaCl_2_, 5 µM N‐ω‐gliadin 34‐mer, or 5 µM N‐α‐gliadin 33‐mer in the presence of primary amines at increasing concentrations (0‐1000 µM), before the addition of TCR‐transfectant cells specific for the DQ2.5‐glia‐α2 or DQ2.5‐glia‐ω2 epitopes. The dose‐response curve fitting to find IC50 was done using the “[Inhibitor] vs. response” equation in GraphPad Prism 8.3.0. To examine whether primary amines directly affect the B cells, TG2‐specific A20 cell expressing DQ2.5 were incubated with primary amines for 30 min and washed, before addition of 2 µM D‐ω‐gliadin 34‐mer, followed by incubation with DQ2.5‐glia‐ω2 TCR transfectants.

## Author contributions

S.F.A. was associated with acquisition of data, analysis and interpretation of data, and drafting of the manuscript. J.S. was associated with analysis and interpretation of data, drafting of manuscript, and critical revision of the manuscript for important intellectual content. M.F.d.P. was associated with acquisition of data and critical revision of the manuscript for important intellectual content. L.M.S. was associated with idea, study concept, and design, drafting of the manuscript, critical revision of the manuscript for important intellectual content, and study supervision.

## Conflict of interest

The authors declare no commercial or financial conflict of interest.

### Peer review

The peer review history for this article is available at https://publons.com/publon/10.1002/eji.202249862.

**Abbreviations**
5‐BP: 5‐biotinamido‐pentylamine/biotinylated cadaverine
Ahx: 6‐aminocaproic acid
BCR: B‐cell receptor
CeD: celiac disease
FCS: fetal calf serum
GDH: glutamate dehydrogenase
HLA: human leukocyte antigen
NADH: nicotinamide adenine dinucleotide hydrogen
TCR: T‐cell receptor
TG2: transglutaminase 2

## Supporting information

Supporting information

## Data Availability

Data sharing not applicable to this article as no datasets were generated or analyzed during the current study.
