# Supplementary material for: Transglutaminase 2 affinity and enzyme‐substrate intermediate stability as determining factors for T‐cell responses to gluten peptides in celiac disease
Source: Eur J Immunol. 2022 Jul 13;52(9):1474–81. doi: 10.1002/eji.202249862 (PMC9545004; doi:10.1002/eji.202249862)
Supplement: Supplementary file 1 — Supporting information [file EJI-52-1474-s001.pdf]

Supporting Information for

Transglutaminase 2 affinity and enzyme-substrate intermediate stability as determining factors for T-cell responses to gluten peptides in celiac disease

Sunniva F. Amundsen[1], Jorunn Stamnaes[1], Marie Fleur du Pré[1,2], Ludvig M. Sollid[1,2]

[1] KG Jebsen Coeliac Disease Research Centre, Institute of Clinical Medicine, University of Oslo, Oslo, Norway

[2] Department Immunology, Oslo University Hospital, Oslo, Norway

Correspondence: Ludvig M. Sollid, Department of Immunology, Oslo University Hospital – Rikshospitalet, 0372 Oslo, Norway. E-mail: l.m.sollid@medisin.uio.no

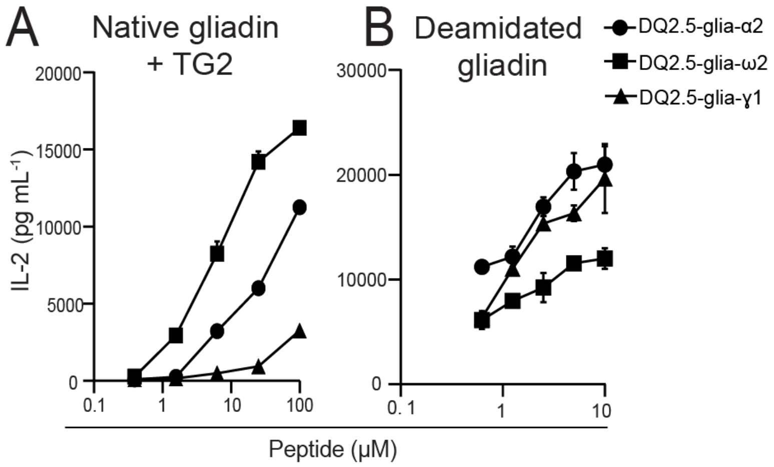

Figure S1. Replicate experiment of Figure 1 showing T-cell activation by B-cell uptake of TG2:gluten complexes or deamidated gluten peptides. A20 cells expressing a TG2-specific BCR and HLA-DQ2.5 were incubated with increasing concentrations of synthetic native or deamidated gluten peptides in presence or absence of active TG2and $CaCl_2$ followed by co-culture with TCR transfectants specific for the respective gluten T-cell epitopes. T-cell activation was assessed by ELISA quantification of IL-2 secreted into the cell culture medium. (A) Activation of TCR-transfectant cells specific for the DQ2.5-glia-α2, DQ2.5-glia-ω2 or DQ2.5-glia-γ1 epitopes after co-culture with TG2-specific A20 cells incubated recombinant TG2, $CaCl_2$ and synthetic native gluten peptides (N-α-gliadin 20-mer, N-ω-gliadin 19-mer and N-γ-gliadin 21-mer). (B) Activation of TCR-transfectant cells specific for the DQ2.5-glia-α2, DQ2.5-glia-ω2 or DQ2.5-glia-γ1 epitopes after co-culture with TG2-specific A20 cells incubated with synthetic deamidated gluten peptides (D-α-gliadin 20-mer, D-ω-gliadin 17-mer and D-γ-gliadin 21-mer). The figure shows mean of sample triplicates +/- SD.

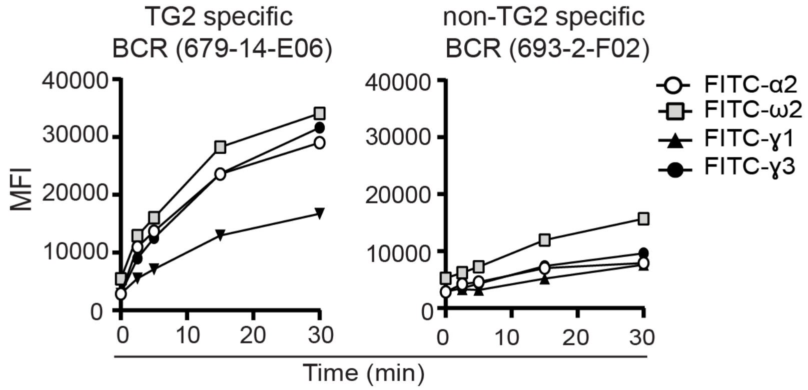

Figure S2 Uptake of TG2:gluten complexes by TG2-specific vs non-TG2-specific B cells. Briefly, A20 cells expressing TG2-specific BCR (679-14-E06) or non-TG2 specific BCR (693-2-F02) were incubated with recombinant TG2 and native FITC 9-mer peptides in the presence of $CaCl_2$. Cell-bound FITC peptide was measured by flow cytometry. Delta (Δ) MFI shown in Figure 3 is calculated by subtracting MFI signal of non-TG2 specific B cells from MFI signal of TG2-specific B cells. The figure shows experimental data from one of three independent experiments.

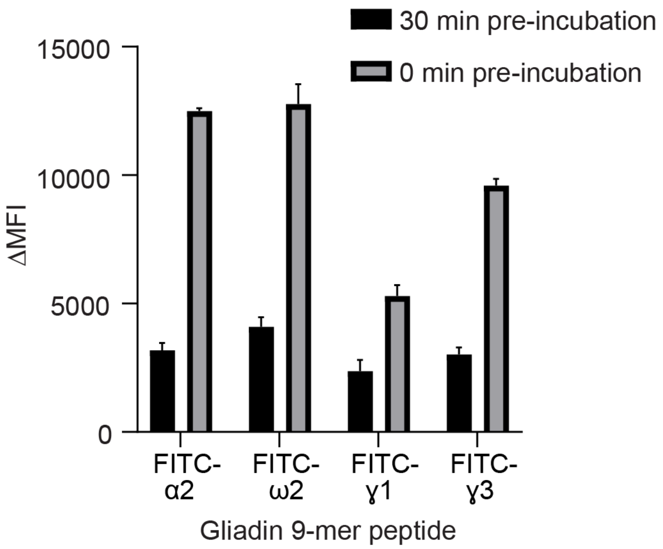

Figure S3 Binding and uptake of fresh vs. pre-formed TG2:gluten complexes by TG2-specific B cells. Recombinant TG2 and native FITC 9-mer peptides were preincubated for 30 min in the presence of $CaCl_2$ to allow for formation of iso-peptide linked complexes. A20 cells expressing TG2 specific BCR (679-14-E06) or non-TG2 specific BCR (639-2-F02) were incubated with pre-incubated TG2:gluten mixture (black bars) or with fresh recombinant TG2, $CaCl_2$ and native FITC-labelled 9-mer peptides (grey bars) for 30min. Cell-bound FITC peptides were measured by flow cytometry. Delta (Δ) MFI was calculated by subtracting MFI of non-TG2 specific B cells from MFI of TG2-specific B cells. The figure shows mean of sample triplicates (+/-SD).

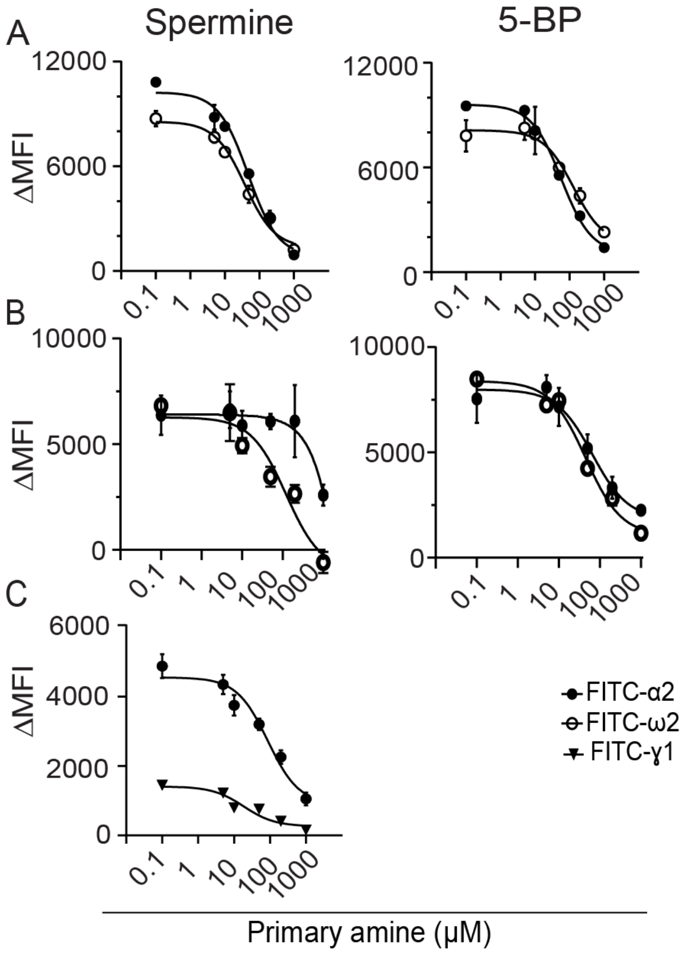

Figure S4 Effect of TG2 secondary substrate on the uptake of TG2:gluten complexes by TG2-specific B cells (Replicate experiments of Figure 4). A20 cells expressing TG2 specific BCR (679-14-E06) or non-TG2 specific BCR (639-2-F02) were incubated for 10 min with recombinant TG2 and FITC-labelled α2, ω2 and γ1 9-mer gliadin peptides in the presence of $CaCl_2$ and increasing concentrations of primary amines. Cell-bound FITC peptides were measured by flow cytometry and delta (Δ) MFI was calculated by subtracting MFI of non-TG2 specific B cells from MFI of TG2-specific B cells. (A, B). Uptake of FITC-α2 and FITC-ω2 in the presence of spermine and 5-BP. A and B are replicate experiment of Figure 4A and B. (C) Uptake of FITC-α2 and FITC-γ1 in the presence of spermine. Replicate experiment of Figure 4C. The figure shows mean of sample triplicates (+/- SD).

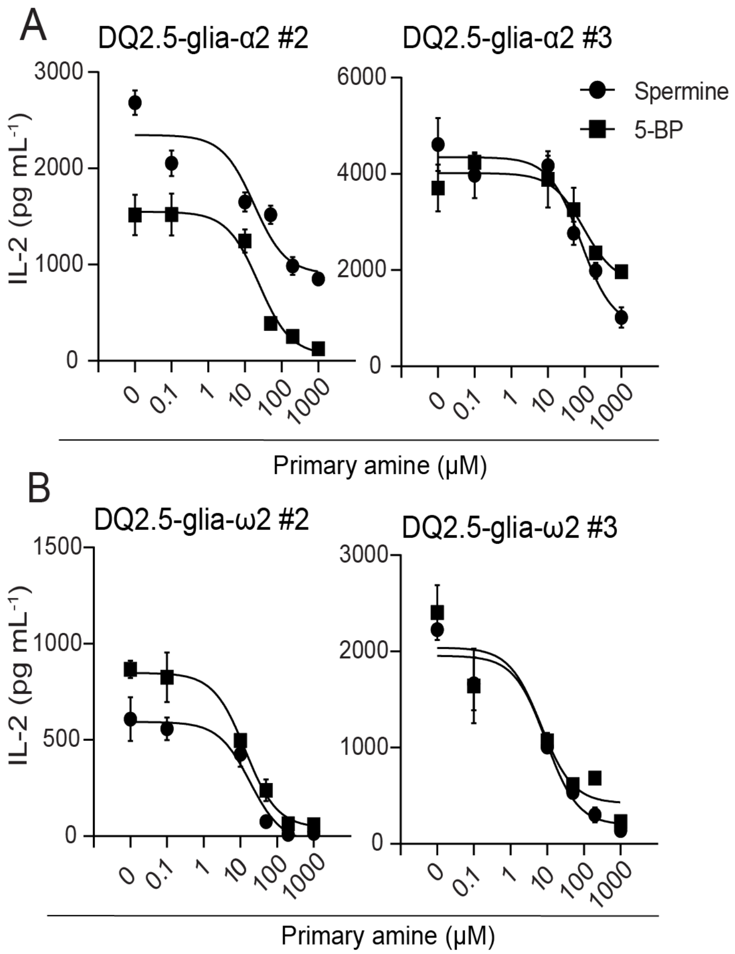

Figure S5 Effect of TG2 secondary substrate on T-cell activation via TG2-specific B cells (Replicate experiments of Figure 5). A20 cells expressing a TG2-specific BCR and HLA-DQ2.5 were incubated with recombinant TG2, N-α-gliadin 33-mer or N-ω-gliadin 34-mer peptides in presence of $CaCl_2$ and increasing concentrations of spermine or 5-BP followed by co-culture with TCR transfectants specific for either the DQ2.5-glia-α2 (A) or DQ2.5-glia-ω2 epitopes (B). T-cell activation was assessed by ELISA quantification of IL-2 secreted into the cell culture medium. The figure shows mean of sample triplicates (+/- SD).

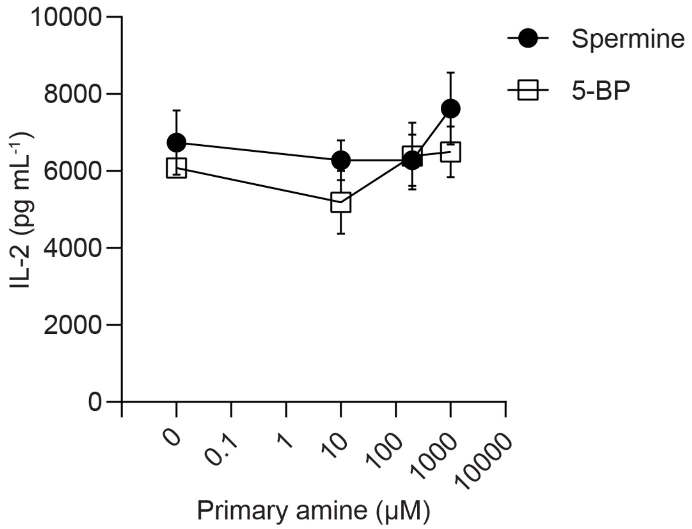

Figure S6 Primary amines used as secondary TG2 substrates have no toxic effect on B cells or T cells in our cellular assays. A20 cells expressing HLA DQ2.5 and TG2-specific BCR were incubated with spermine or 5-BP for 30 min before addition of D-ω-gliadin 34-mer followed by co-culture with TCR transfectants specific for the DQ2.5-glia-ω2 epitope. T-cell activation was assessed by ELISA quantification of IL-2 secreted into the cell culture medium. The figure shows mean of sample triplicates (+/- SD).

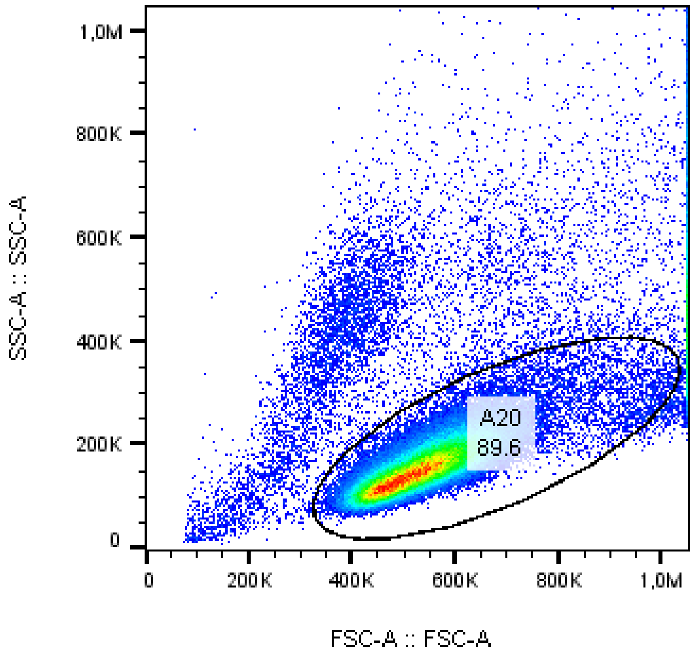

Figure S7: Flow cytometry gating strategy for analysis of A20 B-cells. Cells within the black circle were analyzed.

Table S1:IC50 values for TG2 secondary substrate inhibition TG2:gluten complexes uptake by TG2-specific B cells IC50 values and 95% confidence interval (CI) calculated from experiments shown in Figure 4 and Figure S4 (ND;not determined, IC50 for FITC-ω2 Figure S4 B was not included in calculations of the mean).

**Spermine**	FITC-α2 Figure 4	FITC-α2 Figure S4 A	FITC-α2 Figure S4 B	**FITC-α2 mean**	FITC-ω2 Figure 4	FITC-ω2 Figure S4 A	FITC-ω2 Figure S4 B	**FITC-ω2 mean**	FITC-γ1 Figure 4	FITC-γ1 Figure S4 C	**FITC-γ1 mean**
IC50 (μM)	29.3	47.6	121	**66.1**	14.0	38.2	*2.43E+16*	**26.1**	45.3	18.7	**31.9**
logIC50	1.47	1.68	2.08	**1.74**	1.15	1.58	16.4	**1.36**	1.66	1.27	**1.46**
log 95% CI lower limit	0.911	1.49	1.53	**1.31**	0.93	1.38	*ND*	**1.16**	1.20	0.84	**1.02**
log 95% CI upper limit	2.19	1.864	2.63	**2.23**	1.39	1.79	*ND*	**1.59**	2.08	1.92	**2.0**
**5-BP**											
IC50 (μM)	22.7	57.9	45.4	**42.0**	20.7	130	65.6	**72.0**	*ND*	*ND*	*ND*
logIC50	1.36	1.88	1.66	**1.59**	1.32	2.11	1.82	**1.75**	*ND*	*ND*	*ND*
log 95% CI lower limit	1.04	1.52	1.50	**1.35**	1.09	1.86	1.50	**1.48**	*ND*	*ND*	*ND*
log 95% CI upper limit	1.71	2.00	1.82	**1.85**	1.57	2.38	2.14	**2.03**	*ND*	*ND*	*ND*

Table S2: IC50 values for TG2 secondary substrate inhibition on activation of gluten-specific T cells by TG2-specific B cells IC50 values and 95% confidence interval (CI) calculated from experiments shown in Figure 5 and Figure S5.

**Spermine**	DQ2.5-glia-α2 Figure 5	DQ2.5-glia-α2 Figure S5A	DQ2.5-glia-α2 Figure S5B	**DQ2.5-glia-α2 Mean**	DQ2.5-glia-ω2 Figure 5	DQ2.5-glia-ω2 Figure S5A	DQ2.5-glia-ω2 Figure S5B	**DQ2.5-glia-ω2 Mean**
IC50 (μM)	77.3	17.7	78.9	**58.0**	55.1	18.4	9.13	**27.5**
logIC50	1.89	1.25	1.90	**1.68**	1.74	1.26	0.960	**1.32**
log 95% CI lower limit	1.53	0.638	1.59	**1.25**	1.50	1.03	0.610	**1.04**
log 95% CI upper limit	2.27	1.83	2.22	**2.11**	1.99	1.50	1.26	**1.58**
**5-BP**								
IC50 (μM)	42.7	23.7	93.6	**53.3**	60.7	13.6	6.79	**27.0**
logIC50	1.63	1.37	1.97	**1.66**	1.78	1.13	0.832	**1.25**
log 95% CI lower limit	1.48	1.15	1.54	**1.39**	1.52	0.940	*ND*	**1.23**
log 95% CI upper limit	1.78	1.60	2.43	**1.93**	2.04	1.32	1.36	**1.57**

Table S3: Synthetic gluten peptides used in this study. The “N-“ and “D-“ in front of peptide names denotes native and deamidated versions of peptides, respectively The “q” denotes a pyroglutamate residue. The glutamine/glutamate residue important for the HLA-TCR interaction are given in bold. Residues corresponding to 9-mer core regions of T-cell epitopes are underlined.

Name	Sequence	Source
**FITC-α2**	FITC-Ahx-PQP**Q**LPYPQ	Genscript
**FITC-ω2**	FITC-Ahx-PQP**Q**QPFPW	Genscript
**FITC-γ1**	FITC-Ahx-QQSFP**Q**QQR	Genscript
**FITC-γ3**	FITC-Ahx-QP**Q**QPYPQQ	Genscript
**N-α-gliadin 20-mer**	qLQPFPQP**Q**LPYPQPELPYP	Gift of B. Fleckenstein
**D-α-gliadin 20-mer**	qLQPFPQP**E**LPYPQPELPYP	Gift of B. Fleckenstein
**N-ω-gliadin 19-mer**	PFPQP**Q**QPFPWQPEQPFPQ	Peptide2.0
**D-ω-gliadin 17-mer**	qPQQPFPQP**E**QPFPWQP	GL Biochem
**N-γ-gliadin 21-mer**	YQQLPQPQQPQQSFP**Q**QQRPF	Genscript
**D-γ-gliadin 21-mer**	YQQLPQPEQPQQSFP**E**QERPF	Genscript
**N-α-gliadin 33-mer**	LQLQPFPQP**Q**LPYPQP**Q**LPYPQP**Q**LPYPQPQPF	Genscript
**N-ω-gliadin 34-mer**	QPQQPFPQQPQQPQQPFPQP**Q**QPFPWQPQQPFPQ	GL Biochem
**D-ω-gliadin 34-mer**	QPQQPFPEQPQQPEQPFPQP**E**QPFPWQPEQPFPQ	Genscript
